# Serum vitamin D and obesity among US adolescents, NHANES 2011–2018

**DOI:** 10.3389/fped.2024.1334139

**Published:** 2024-05-21

**Authors:** Zisu Chen, Xiaojin Qiu, Qiong Wang, Jing Wu, Min Li, Wenquan Niu

**Affiliations:** ^1^ Department of Pediatrics, Beijing Hospital of Traditional Chinese Medicine, Capital Medical University, Beijing, China; ^2^ Department of Pediatrics, China-Japan Friendship Hospital, Beijing, China; ^3^ Graduate School, Beijing University of Chinese Medicine, Beijing, China; ^4^ Center for Evidence-Based Medicine, Capital Institute of Pediatrics, Beijing, China

**Keywords:** 25-hydroxyvitamin D, obesity, HOMA-IR, adolescents, NHANES

## Abstract

**Background and objectives:**

Childhood obesity is highly prevalent worldwide. We aimed to assess whether serum 25-hydroxyvitamin D was associated with general/central obesity among US adolescents, and further to explore the mediatory impact of homeostasis model assessment of insulin resistance (HOMA-IR) on this association.

**Methods:**

This study is cross-sectional in design. Study adolescents were enrolled from the National Health and Nutrition Examination Survey (NHANES), 2011–2018. Serum 25-hydroxyvitamin D categories associated with general (indexed by body mass index) and central (indexed by waist circumference to height ratio) obesity were regressed. The possible mediatory effect of HOMA-IR on this association was explored. The nonlinear and dose-response association was examined by restricted cubic spline (RCS) test.

**Results:**

Total 2,696 adolescents were eligible for inclusion, and the mean age of all adolescents was 15.4 years. Overall, the percentage of general and central obesity was 38.0% and 38.6%, respectively. Compared with adolescents with sufficient vitamin D, adolescent with deficient and insufficient vitamin D intake were associated with general obesity and central obesity; fully-adjusted OR for general obesity was 1.602 (95% CI: 1.161–2.211) and 1.659 (1.385–1.986), and fully-adjusted OR for central obesity was 2.025 (1.445–2.837) and 1.557 (1.287–1.884), respectively, while there was no observable significance in adolescents with possibly harmful vitamin D. The proportion mediated by HOMA-IR was estimated to be 31.7% for global obesity and 50.3% for central obesity (both *P* < 0.05). More stratified analyses were presented, and identified that the association with general obesity was particularly present among Mexican American, while with central obesity among Non-Hispanic Black adolescents.

**Conclusions:**

Our findings indicate that deficient or insufficient 25-hydroxyvitamin D concentrations were associated with the significant risk of general and central obesity among US adolescents, and approximately 30% and 50%, respectively, of these associations were mediated by HOMA-IR.

## Introduction

Childhood obesity is a prevalent public health issue worldwide (1). Statistics from the World Health Organization show that the global prevalence of overweight or obesity in children and adolescents increased from 5% in 1975 to 18% in 2016. In the United States (US), 34.5% of adolescents 12–19 years of age were overweight or obese (2). Generally, obesity in adolescence persists into adulthood, and it can trigger the development of chronic diseases (such as diabetes and cardiovascular disease) and premature death, as well as psychological social and emotional well-being and self-esteem issues (3–5). Hence, identification of the risk factors of childhood obesity may enhance knowledge on underlying causes and inform therapeutic strategies toward a more effective prevention of obesity and resultant complications.

It is increasingly recognized that vitamin D is an essential nutrient responsible for health maintenance of bones and muscles (6). Vitamin D deficiency, as reflected by low 25-hydroxyvitamin D concentrations, is highly prevalent in children with obesity (7). Vitamin D is fat-soluble and can be affected by diet and sunlight, as well as obesity and sedentarism. Observational studies have shown that vitamin D deficiency was associated with general and central obesity in adults (7); however, evidence is sparse in children and adolescents. Biologically, vitamin D can regulate cell differentiation and growth via binding to vitamin D receptor in most body cells (8). Cell-signaling mechanisms linking vitamin D to obesity are multifaceted, possibly involving matrix metalloproteinases, mitogen-activated protein kinase pathways, reactive oxygen species, and nitric oxide synthase (8). In addition, vitamin D, from either exogenous or endogenous sources, becomes sequestered within adipose tissues (9), and excess adiposity may directly affect its bioavailability (10). Above lines of evidence collectively inspire us to speculate that vitamin D deficiency is a potential risk trigger for obesity in children and adolescents. However, the relationship between them can be bidirectional, since obesity can also lead to vitamin D deficiency (11).

Moreover, the relation between 25-hydroxyvitamin D and insulin resistance has been widely assessed (12–15). For example, in Turkey, serum 25-hydroxyvitamin D was found to be negatively correlated with insulin and homeostasis model assessment of insulin resistance (HOMA-IR) in children 5–17 years of age (16). In prepubertal Chilean children, there was an inverse association of 25-hydroxyvitamin D with adiposity and insulin resistance indicators (17). Given the close relation between insulin resistance and obesity, it is reasonable to speculate the association between 25-hydroxyvitamin D and obesity might be mediated through insulin resistance.

To address above two speculations, we aimed to assess whether serum 25-hydroxyvitamin D was associated with general/central obesity among US adolescents, and further to explore the mediatory impact of HOMA-IR on this association.

## Methods

### Data source and study subjects

This study used data from the National Health and Nutrition Examination Survey (NHANES), which is an ongoing two-year-cycle nationally representative survey in the US to monitor the health and nutritional status of adults and children. Detailed design and data collection of NHANES has been reported previously (18–20). All survey protocols were approved by the research ethics review board at the National Center for Health Statistics, and written informed consent was obtained from all respondents.

**Figure 1:**
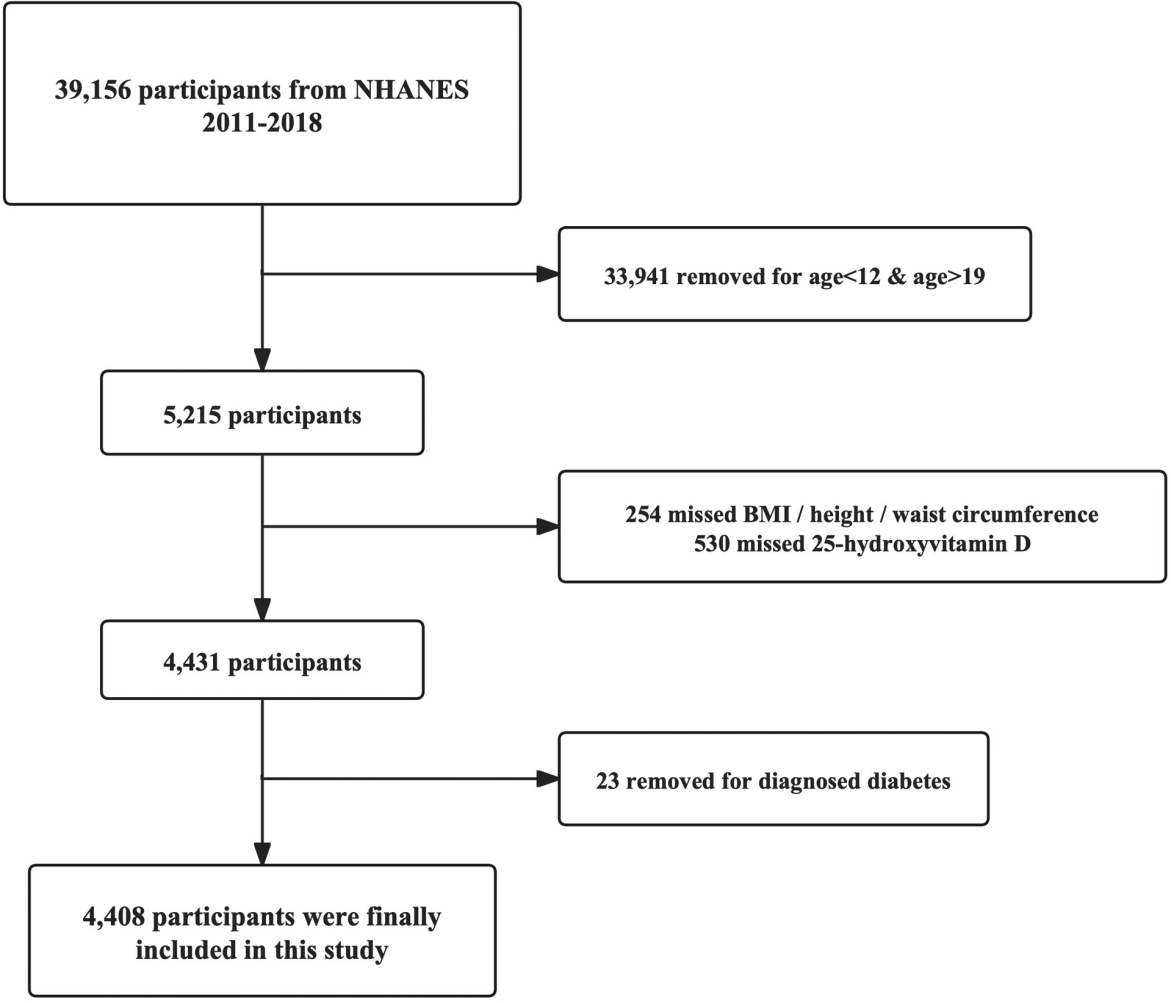
Selection flowchart of study adolescents in this study.

All subjects were selected from respondents attending 4 NHANES cycles, 2011–2018. Only adolescents 12–18 years of age were eligible for inclusion, with complete data on serum 25-hydroxyvitamin D concentrations and body mass index (BMI) or waist circumference. Adolescents were excluded if they had a diagnosis of diabetes mellitus. In total, 2,696 adolescents were finally analyzed in this study, and the selection process is illustrated in Figure 1.

### General/central obesity

In this study, both general and central obesity were assessed. Specifically, body mass index (BMI) was chosen as an indicator of general obesity, and waist circumference to height ratio (WHtR) as an indicator of central obesity (21). Data on body measures were collected from the Mobile Examination Center (MEC) by trained health technicians.

BMI is calculated as weight in kilograms divided by height in meters squared. The classification are based on the Centers for Disease Control and Prevention's sex-specific 2,000 BMI-for-age growth charts for the United States. Overweight is BMI 85th percentile to <95th percentile. Obesity is BMI ≥ 95th percentile. Overweight and obesity was combined to define general obesity (18). WHtR is calculated as waist circumference in centimeters divided by height in centimeters. Central obesity is defined as WHtR of at least 0.5, as proposed previously (22).

### Serum 25-hydroxyvitamin D

Serum 25-hydroxyvitamin D concentrations were assayed by the standardized liquid chromatography-tandem mass spectrometry (LC-MS/MS) method. According to the CDC (23), serum 25-hydroxyvitamin D concentrations less than 30 nmol/L were considered deficient, 30–50 nmol/L insufficient, 50–125 nmol/L sufficient, and exceeding 125 nmol/L possibly harmful.

### HOMA-IR

Fasting glucose and insulin blood samples were collected by trained phlebotomists in the MEC. HOMA-IR were calculated by the formulas: fasting glucose (mmol/L)*fasting insulin (µU/ml)/22.5. HOMA-IR used for the mediation analysis were treated as categorical using the cut-offs for age and gender (24).

### Other factors

During the in-home interview, demographic information was collected through the Computer-Assisted Personal Interview system, including age, sex, race/ethnicity, and poverty income ratio (PIR). PIR was calculated by dividing family income to the poverty guidelines specific for each survey year, and 2% was set as the threshold below which difficult financial conditions were assumed (25).

Dietary energy and vitamin D intakes were estimated using the mean of two 24-h dietary recall interviews from the Dietary Interview Questionnaire (19). Physical activity was obtained from the NHANES Physical Activity Questionnaire (PAQ). Different types of physical activities have different MET values and NHANES provides recommended MET values for sports of different types. Physical activity was calculated according to the following formula: physical activity (MET-h/week) = MET × weekly frequency × duration of each physical activity (20).

All variables under study can be found on the official NHANES website https://www.cdc.gov/nchs/nhanes/index.htm.

### Statistical analyses

The Shapiro–Willk test was used to test the normality of distribution of continuous data, and according to its results, continuous variables were represented as median (interquartile range) because of skewed distribution. Categorical variables were represented as numbers and percentages. The Kruskal–Wallis test and *χ*^2^ test were used to compare the differences between groups according to vitamin D status. If significant, pairwise post-hoc Dunn's test. The association of serum 25-hydroxyvitamin D on a categorical scale with general and central obesity was assessed using the Logistic regression analyse. Three models were built. Model 1 did not adjust for any covariates. In model 2, we adjusted for age, sex, race/ethnicity, and PIR; In model 3, we further adjusted for energy intake and physical activity. We conducted stratified analyses according to age at baseline, sex, race, PIR and physical activity in the logistic regression models. Tests for interaction were performed by adding interaction terms in Model 3.

Besides overall association, subgroup analyses were also performed according to age, sex, race/ethnicity, PIR, and physical activity, respectively. The Sobel-Goodman mediation test was used to examine whether HOMA-IR can mediate the association of 25-hydroxyvitamin D with general and central obesity. Nonlinear and dose-response relation was examined by restricted cubic spline (RCS) curve. The possibility of unmeasured confounding factors was evaluated using the *E*-values (26).

Two-sided *P* below 0.05 was considered statistically significant. Data were analyzed using Stata software version 17 (StataCorp LP, TX, USA) and R programming environment version 4.2.3 available at website https://www.r-project.org/.

## Results

### Baseline characteristics

The baseline characteristics of 2,696 study adolescents by serum 25-hydroxyvitamin D categories are presented in Table 1. The mean age of all adolescents was 15.4 years, and boys accounted for 53.7%. The percentage of general and central obesity was 38.0% and 38.6%, respectively. Besides fasting glucose, all baseline characteristics differed significantly across 25-hydroxyvitamin D categories (*P* < 0.001).

**Table 1:** Baseline characteristics of study adolescents aged 12–19 years from the NHANES, 2011–2018.

Characteristics	Total	Serum 25-hydroxyvitamin D (nmol/L)				*P*
		Sufficient (50–125)	Deficient (<30)	Insufficient (30–49)	Possibly harmful (>125)	
Sample size	2,696	1,638	187	855	16	
Age, years	15.4 (13.0, 17.0)	15.0 (13.0, 17.0)	16.0 (14.0, 18.0)^‡^	16.0 (14.0, 17.0)*	17.0 (14.5, 18.0)^†^	<0.001
Sex						<0.001
Boys	1,448 (53.7%)	943 (65.1%)	73 (5.0%)*	428 (29.6%)*	4 (0.3%)^‡^	
Girls	1,248 (46.3%)	695 (55.7%)	114 (9.1%)*	427 (34.2%)*	12 (1.0%)^‡^	
Race/ethnicity						<0.001
Mexican American	560 (20.8%)	313 (55.9%)	26 (4.6%)*	220 (39.3%)*	1 (0.2%)	
Other hispanic	298 (11.1%)	184 (61.8%)	15 (5.0%)*	99 (33.2%)*	0 (0.0%)	
Non-hispanic white	725 (26.9%)	634 (87.4%)	4 (0.6%)*	75 (10.3%)*	12 (1.7%)	
Non-hispanic black	665 (24.7%)	239 (35.9%)	108 (16.2%)*	317 (47.7%)*	1 (0.2%)	
Other races	448 (16.6%)	268 (59.8%)	34 (7.6%)*	144 (32.2%)*	2 (0.4%)	
Poverty income ratio						<0.001
<2	1,596 (59.2%)	882 (55.3%)	132 (8.3%)*	575 (36.0%)*	7 (0.4%)	
≥2	1,100 (40.8%)	756 (68.7%)	55 (5.0%)*	280 (25.5%)*	9 (0.8%)	
Physical activity^b^						<0.001
Low	1,456 (54.0%)	843 (57.9%)	129 (8.9%)*	480 (33.0%)	4 (0.2%)	
High	1,240 (46.0%)	795 (64.1%)	58 (4.7%)*	375 (30.2%)	12 (1.0%)	
Energy intake, kcal^a^	2,006 (1,445, 2,410)	1,924 (1,499, 2,464)	1,714 (1,332, 2,169)*	1,830 (1,390, 2,332)*	2,007 (1,725, 2,411)	
Vitamin D intake, mcg^a^	5.0 (2.0, 6.7)	4.6 (2.4, 7.5)	2.1 (1.1, 4)*	3.5 (1.5, 5.6)*	5.8 (2.7, 8.0)	<0.001
25-hydroxyvitamin D, nmol/L	56.9 (43.1, 67.6)	63.8 (57.1, 75.4)	24.1 (21.9, 27.3)*	41.9 (36.4, 46.4)*	135.5 (130.0, 143.0)*	<0.001
Fasting glucose, mmol/L	5.2 (4.9, 5.5)	5.2 (4.9, 5.5)	5.2 (4.9, 5.5)	5.2 (4.9, 5.5)	5.3 (4.9, 5.3)	0.8726
Insulin, µU/ml	14.2 (7.2, 16.3)	9.9 (6.8, 14.4)	14.4 (7.9, 20.2)*	11.8 (7.8, 18.6)*	7.6 (5.9, 14.2)	<0.001
HOMA-IR^c^	3.4 (1.6, 3.9)	2.3 (1.56, 3.4)	3.1 (1.6, 4.4)^‡^	2.7 (1.8, 4.5)*	1.7 (1.4, 3.3)	<0.001
BMI (kg/m^2^)	24.2 (19.8, 27)	22.2 (19.6, 25.9)	23.2 (20.0, 30.2)	23.6 (20.2, 29.1)	21.9 (19.4, 23.7)	<0.001
Non-overweight	1,671 (62.0%)	1,087 (65.1%)	106 (6.3%)*	466 (27.9%)*	12 (0.7%)	
Overweight	458 (17.0%)	281 (61.4%)	27 (5.9%)*	148 (32.3%)*	2 (0.4%)	
Obesity	567 (21.0%)	270 (47.6%)	54 (9.5%)*	241 (42.5%)*	2 (0.4%)	
WHtR^c^	0.5 (0.4, 0.5)	0.5 (0.4, 0.5)	0.5 (0.4, 0.6)*	0.5 (0.4, 0.6)*	0.5 (0.4, 0.5)	<0.001
Normal	1,656 (61.4%)	1,082 (65.3%)	93 (5.6%)*	469 (28.3%)	12 (0.8%)	
Central obesity	1,040 (38.6%)	556 (53.5%)	94 (9.0%)*	386 (37.1%)	4 (0.4%)	

HOMA-IR, homeostatic model assessment of insulin resistance; WHtR, waist circumference to height ratio; BMD, bone mineral density; BMI, body mass index.

Continuous data are represented as median (interquartile range). Categorical data are expressed in percentage.

^a^ Vitamin D intake and Energy include two 24-h dietary recall interviews.

^b^ Physical activity (MET-h/wk) = MET × weekly frequency × duration of each physical activity.

Covariates labeled by letters *, ^†^, ^‡^ showed statistically significant differences (paired) through the vitamin D categories using *post hoc* pairwise comparison, Dunn's Test.

^*^ *P* < 0·001.

^†^ *P* < 0·05.

^‡^ *P* < 0·01.

Compared with adolescents with sufficient vitamin D, adolescents who had deficient and insufficient serum vitamin D were more likely to be older, girls, non-Hispanic Black (predominantly, both deficient and insufficient) and Mexican American (only insufficient), with low physical activity and PIR, with lower vitamin D and total energy intakes, with higher insulin levels and HOMA-IR, and with global and central obesity.

### Overall analyses

Table 2 shows the association of serum 25-hydroxyvitamin D with general and central obesity. Taking adolescents with sufficient 25-hydroxyvitamin D as a reference, the risk for both general and central obesity was significantly increased in adolescents with deficient and insufficient 25-hydroxyvitamin D before and after adjusting for confounding factors. In adolescents with deficient and insufficient vitamin D, fully-adjusted OR associated with general obesity was 1.602 (95% CI: 1.161–2.211) and 1.659 (1.385–1.986), with central obesity was 2.025 (1.445–2.837) and 1.557 (1.287–1.884), respectively. By contrast, no hint of statistical significance was seen in adolescent with possibly harmful 25-hydroxyvitamin D.

**Table 2:** Association of 25-hydroxyvitamin D with general and central obesity.

Serum 25-hydroxyvitamin D	General obesity			Central obesity		
	Model 1	Model 2	Model 3	Model 1	Model 2	Model 3
*N*	2,696	2,696	2,696	2,696	2,696	2,696
Sufficient	(Ref.)	(Ref.)	(Ref.)	(Ref.)	(Ref.)	(Ref.)
Deficient	1.650 (1.224, 2.225)**	1.644 (1.198, 2.256)**	1.602 (1.161, 2.211)**	1.967 (1.451, 2.667)***	2.146 (1.542, 2.988)***	2.025 (1.445, 2.837)***
Insufficient	1.733 (1.471, 2.041)***	1.685 (1.410, 2.012)***	1.659 (1.385, 1.986)***	1.602 (1.352, 1.897)***	1.602 (1.328, 1.933)***	1.557 (1.287, 1.884)***
Possibly harmful	0.671 (0.218, 2.064)	0.706 (0.228, 2.192)	0.767 (0.246, 2.388)	0.649 (0.208, 2.021)	0.555 (0.176, 1.757)	0.607 (0.191, 1.927)

Ref., reference. Data are represented as odds ratio (95% confidence interval).

Model 1: no adjustment.

Model 2: adjustment for age, sex, race/ethnicity, and poverty income ratio.

Model 3: adjustment for age, sex, race/ethnicity, poverty income ratio, vitamin D intake, energy, physical activity.

* *P* < 0.05.

** *P* < 0.01.

*** *P* < 0.001.

**Figure 2:**
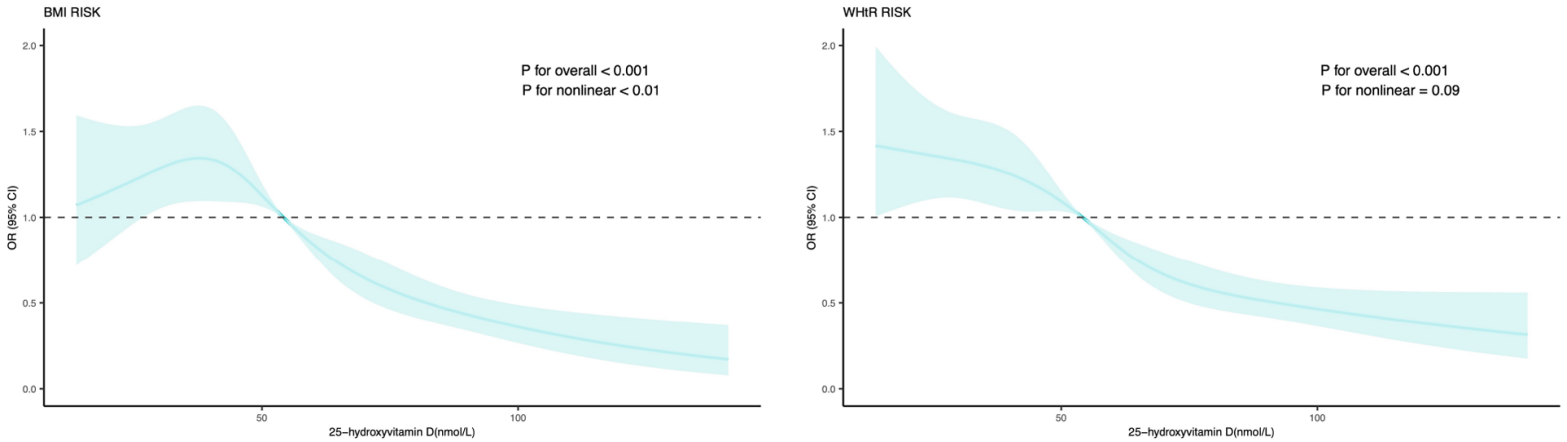
Dose-response relation of serum 25-hydroxyvitamin D with general and central obesity. Effect-size estimates were calculated after adjusting for age, sex, race/ethnicity, poverty income ratio, vitamin D intake, energy, physical activity. BMI, body mass index; WHtR, waist circumference to height ratio; OR, odds ratio; 95% CI, 95% confidence interval.

The dose-response relation of serum 25-hydroxyvitamin D with general and central obesity was also explored (Figure 2), and it was statistically significant at a level of 1‰. Based on the dose-response, vitamin D below about 55 nmol/L increases the risk of general and central obesity.

### Mediatory effect

Table 3 shows the mediation effect of HOMA-IR on the association of serum 25-hydroxyvitamin D with general and central obesity. Total, natural direct, and natural indirect effects were explored, with statistical significance at a level of 5%. The proportion mediated by HOMA-IR reached as high as 31.7% for global obesity and 50.3% for central obesity (both *P* < 0.01).

**Table 3:** Mediatory effect of HOMA-IR on the association of serum 25-hydroxyvitamin D with general and central obesity.

Items	Statistics	General obesity	Central obesity
Total effect	β	0.095	0.033
	Lower	0.046	0.004
	Upper	0.143	0.063
	*P*	<0.001	<0.05
Natural direct effect	β	0.065	0.017
	Lower	0.022	−0.008
	Upper	0.107	0.041
	*P*	<0.01	0.189
Natural indirect effect	β	0.030	0.017
	Lower	0.007	0.004
	Upper	0.053	0.030
	*P*	<0.05	<0.05
Proportion eliminated		31.7%	50.3%
*P*-value		<0.05	<0.05

HOMA-IR, homeostatic model assessment of insulin resistance.

*P* was calculated after adjusting for age, sex, race/ethnicity, poverty income ratio, vitamin D intake, energy, physical activity.

### Subgroup analyses

To further account for possible confounding effects, subgroup analyses were conducted according to age, sex, race/ethnicity, PIR, and physical activity after adjusting for confounders and taking sufficient 25-hydroxyvitamin D as a reference (Table 4). The associations between vitamin D and obesity were not significantly modified by age, sex, race/ethnicity, PIR, and physical activity (all *P*-interaction >0.05).

**Table 4:** Subgroup analyses on the association of serum 25-hydroxyvitamin D with general and central obesity.

Subgroups	General obesity				Interaction *P*-value	Central obesity				Interaction *P*-value
	Sufficient	Deficient	Insufficient	Possibly harmful		Sufficient	Deficient	Insufficient	Possibly harmful	
Age					0.628					0.920
12–15 years	(Ref.)	1.126 (0.692, 1.832)	1.405 (1.085, 1.819)**	0.970 (0.172, 5.455)		ref.	1.585 (0.969, 2.593)	1.496 (1.146, 1.952)**	0.983 (0.172, 5.632)	
16–18 years	(Ref.)	1.866 (1.167, 2.982)**	1.775 (1.348, 2.337)***	0.605 (0.125, 2.939)		ref.	2.591 (1.612, 4.163)***	1.676 (1.271, 2.210)***	0.430 (0.089, 2.089)	
Sex					0.814					0.512
Boys	(Ref.)	1.742 (1.043, 2.908)*	1.506 (1.163, 1.950)**	2.433 (0.337, 17.58)		ref.	2.239 (1.328, 3.775)**	1.480 (1.133, 1.935)**	0.857 (0.087, 8.439)	
Girls	(Ref.)	1.234 (0.786, 1.936)	1.642 (1.247, 2.161)***	0.443 (0.095, 2.073)		ref.	1.890 (1.207, 2.958)**	1.644 (1.248, 2.165)***	0.561 (0.147, 2.141)	
Race/ethnicity					0.295					0.248
Mexican American	(Ref.)	3.372 (1.374, 8.276)**	2.000 (1.385, 2.888)***	NA		ref.	2.679 (1.059, 6.775)*	1.792 (1.240, 2.589)**	NA	
Other hispanic	(Ref.)	1.814 (0.580, 5.671)	1.489 (0.876, 2.531)	NA		ref.	2.093 (0.625, 7.014)	1.641 (0.965, 2.790)	NA	
Non-hispanic white	(Ref.)	0.577 (0.0,580, 5.741)	1.618 (0.976, 2.684)	0.190 (0.024, 1.501)		ref.	2.027 (0.262, 15.690)	1.402 (0.841, 2.339)	NA	
Non-hispanic black	(Ref.)	1.384 (0.844, 2.269)	1.513 (1.053, 2.175)**	NA		ref.	2.968 (1.774, 4.966)***	2.011 (1.359, 2.976)***	NA	
Other races	(Ref.)	0.765 (0.308, 1.899)	1.436 (0.913, 2.258)	NA		ref.	1.070 (0.474, 2.420)	1.173 (0.739, 1.862)	2.292 (0.135, 38.990)	
Poverty income ratio					0.177					0.327
<2	(Ref.)	1.192 (0.795, 1.789)	1.440 (1.143, 1.815)**	1.453 (0.316, 6.687)		ref.	2.041 (1.352, 3.081)***	1.590 (1.253, 2.017)***	0.651 (0.119, 3.545)	
≥2	(Ref.)	2.252 (1.238, 4.097)**	1.798 (1.307, 2.474)***	0.312 (0.038, 2.546)		ref.	2.176 (1.192, 3.973)*	1.513 (1.096, 2.089)*	0.616 (0.123, 3.090)	
Physical activity					0.777					0.862
Low	(Ref.)	1.509 (1.000, 2.277)	1.671 (1.300, 2.147)***	0.789 (0.080, 7.756)		ref.	2.088 (1.378, 3.162)***	1.627 (1.262, 2.099)***	0.495 (0.050, 4.923)	
High	(Ref.)	1.435 (0.799, 2.576)	1.471 (1.107, 1.955)**	0.712 (0.188, 2.697)		ref.	2.119 (1.173, 3.827)*	1.505 (1.125, 2.013)**	0.712 (0.187, 2.709)	

Ref., reference; NA, not available. Data are represented as odds ratio (95% confidence interval).

*P* was calculated after adjusting for age, sex, race/ethnicity, poverty income ratio, vitamin D intake, energy, physical activity.

* *P* < 0.05.

** *P* < 0.01.

*** *P* < 0.001.

By age, the risk for general and central obesity conferred by deficient and insufficient serum 25-hydroxyvitamin D was more obvious in adolescents aged 16–18 years than 12–15 years. By sex and race/ethnicity, deficient serum 25-hydroxyvitamin D was associated with general obesity in boys but not in girls, and in adolescents of Mexican American descent but not of others. Deficient and insufficient serum 25-hydroxyvitamin D concentrations were associated with the significant risk of central obesity in both sexes and in adolescents of Mexican American and non-Hispanic Black descents.

PIR and physical activity did not significantly influence the risk for centripetal obesity among vitamin D both insufficient and deficient adolescents and the risk for global obesity among vitamin D insufficient adolescents, but among vitamin D deficient adolescents the risk for global obesity was statistically significant only among those with high PIR (note: for other vitamin D deficient PIR and physical activity subcategories, the risk was also high but did not rich significance).

Still, there was no observable significance across all subgroups in adolescents with probably harmful 25-hydroxyvitamin D.

The stratified estimated probabilities for general and central obesity with increasing serum 25-hydroxyvitamin D concentrations are illustrated in Supplementary Figure S1.

### Unmeasured confounding evaluation

The possibility of unmeasured confounders was evaluated by using the *E*-values (Table 5). Relative to adolescents with sufficient 25-hydroxyvitamin D, *E*-values were estimated to be 1.846 and 1.897 in adolescent with deficient and insufficient 25-hydroxyvitamin D for general obesity, and 2.199 and 1.804 for central obesity. As these *E*-values were larger or almost equivalent to effect-size estimates of serum 25-hydroxyvitamin D associated with general and central obesity, the likelihood for the existence of unmeasured confounders was relatively low.

**Table 5:** *E*-values for the association of serum 25-hydroxyvitamin D with general and central obesity.

25-hydroxyvitamin D	General obesity	Central obesity
Sufficient	(Ref.)	(Ref.)
Deficient	1.846 (1.366)	2.199 (1.695)
Insufficient	1.897 (1.633)	1.804 (1.525)

Ref., reference. Data in parentheses represents the low limit of effect-size estimates.

## Discussion

This study aimed to assess whether serum 25-hydroxyvitamin D was associated with general/central obesity among US adolescents, and explore the mediatory impact of HOMA-IR on this association. The key findings of this study are that deficient and insufficient 25-hydroxyvitamin D concentrations were associated with general and central obesity among the USA adolescents, and approximately 30% and 50%, respectively, of these associations were mediated by HOMA-IR. To our knowledge, this is the thus far the first study that has explored the mediatory effect of HOMA-IR on the association between vitamin D and obesity in adolescents.

Some studies have reported that vitamin D was significantly associated with obesity in children and adolescents, whereas others failed to support this claim. For instance, in 494 children and adolescents from Colombia, insufficient 25-hydroxyvitamin D was associated with over 70% increased risk of overweight or obesity (27). In support of this association, a study of 2,680 children and adolescents from China demonstrated over 90% increased risk of overweight or obesity was attributable to deficient and insufficient vitamin D (28). By contrast, in 2,818 children and adolescents from China, vitamin D status was not associated with obesity (29). Another studies in 1,090 adolescents from Iran also failed to document any significant association between 25-hydroxyvitamin D and anthropometric measures (30). The reasons behind this controversy are manifold, likely relating to diverse origins of study populations, differing age groups, different eligibility criteria, low statistical power, or insufficient consideration of confounding factors. Bearing these reasons in mind, we employed the high-quality NHANES data, with continuous quality assurance and quality control, and among 2,696 adolescents 12–18 years of age assessed the association of 25-hydroxyvitamin D, the major circulating form of vitamin D, with both general and central obesity after considering a wide panel of confounders. It is worth noting that deficient and insufficient 25-hydroxyvitamin D concentrations were significantly and independently associated with the increased risk of both general and central obesity, consistent with the results of a recent meta-analysis by Fiamenghi and Mello (31). Nevertheless, we agree that much needs to be done before translating our findings into practice from both clinical and public health standpoints.

There are two possible mechanisms underlying the association between vitamin D and obesity. One is that vitamin D can directly influence fat accumulation, re-distribution and metabolism since it may regulates lipolysis and lipid synthesis and improve adipose tissue inflammation. Another possible mechanism is that vitamin D may indirectly contribute to weight gain and fat accumulation by regulating parathyroid hormone, calcium, and leptin (8, 32–34).

Based on above epidemiological and biological evidence, it is expected that proper supplementation of vitamin D, if involved, can form an effective preventive strategy for the onset and progression of obesity in adolescents. However, the evidence for the beneficial effect of vitamin D supplementation on BMI is conflicting, with no significant weight reduction observed in overweight and obese subjects after supplementation (35–37). On the other side, some authors observed a reduction in truncal subcutaneous fat and reversal to normoglycemia in the overweight/obese subjects after supplementation (35, 37–39). Therefore, this hypothesis requires more rigorous and evidence-based experiments to be designed for further research.

Since there is much controversy on the cause-effect direction of the association of general and central obesity with vitamin D (11), we performed supplementary analyses in the opposite way: the influence of general and central obesity on serum 25-hydroxy vitamin D levels and the mediatory impact of HOMA-IR on this association (Supplementary Material Tables S1,S2). The results were quite similar, both types of obesity were associated with low vitamin D levels, with HOMA-IR explaining significantly the association of central obesity with vitamin D levels, while less explaining the association of general obesity with vitamin D levels. Potential causative mechanisms for the low serum 25-hydroxyvitamin D concentrations in general/central obesity include volumetric dilution, sequestration into adipose tissue, limited sunlight exposure of overweight/obese people, and reduced vitamin D synthesis and activation in the skin, adipose tissue and the liver (35, 39, 40).

Besides the convincing association between vitamin D and obesity, we explored the possible mediatory effect of insulin resistance on this association in this study. Growing data indicate the close relation between 25-hydroxyvitamin D and HOMA-IR, both of which were reported to be significantly associated with obesity in children and adolescents (41–43). Yet, a literature search has failed to reveal any evidence on the mediatory of HOMA-IR on the vitamin D-obesity association. To fill this gap in knowledge, we interestingly found that over 30% and 50% of these associations, respectively, for general and central obesity, can be mediated by HOMA-IR. The proposed mechanisms for the role of 25-hydroxyvitamin D in improving insulin resistance in obesity include reducing inflammation, enhancing peripheral and hepatic glucose uptake, and regulating insulin synthesis and secretion by pancreatic β cells through direct and indirect pathways (44). In addition, we also noticed that association with central obesity was lost when HOMA-IR was taken into account (which means that insulin resistance mediated completely this association), while with general obesity remained (insulin resistance did not mediate completely the association with general obesity). This is in accordance with the theory of dilution of vitamin D in fat tissue (11). However, association with insulin resistance has an additional, independent influence, apart from fat tissue dilution. This means that both mechanisms are generally included in general obesity, while in central obesity, the mechanism of insulin resistance predominates. Also in our supplementary study, approximately 28.6% and 36.6%, respectively, of these associations were mediated by HOMA-IR. The presence of vitamin D receptors and vitamin D metabolizing enzymes in insulin-sensitive organs suggests that vitamin D may be involved in glucose and lipid metabolism and may be associated with insulin sensitivity. Several studies support a role for vitamin D in regulating glucose and lipid metabolism in several insulin-sensitive tissues, including adipose tissue, skeletal muscle, liver, and pancreatic insulin secretion (14, 45). As well, a potential role for vitamin D in intestinal barrier function and metabolism has been proposed (46). However, the opposite direction can exist in the relation insulin resistance/diabetes and vitamin D metabolism. For example, Aatsinki et al. (47) showed that vitamin D metabolizing enzymes are altered in experimentally induced diabetic states (both type 1 and type 2 diabetes), leading to the repression of vitamin D bioactivation and induction of deficiency, by the mechanisms that include the peroxisome proliferator-activated receptor-gamma coactivator 1-α and estrogen-related receptor α (PGC-1α-ERRα), and the glucocorticoid receptor pathways (47).

To further investigate the association between 25-hydroxyvitamin D and obesity/central obesity, we performed subgroup analyses by age, sex, race, PIR, and PA. From the results, elder age, male, Mexican American and Non-Hispanic Black, poverty and low exercise were significantly associated with higher odds of obesity/central obesity. Age and ethnicity have been shown to affect serum vitamin D status (48, 49). Among them, in Mexican Americans and non-Hispanic blacks vitamin D levels were significantly associated with the prevalence of obesity. Moreover, there was an interesting finding in our study that the association with general obesity was particularly present among Mexican Americans, while with central obesity among Non-Hispanic Black adolescents. The explanations for such findings among these two populations could be the darker color of skin (50, 51), the increased prevalence of obesity and IR (52, 53), and specific lifestyle factors (exposure to sun, physical activity, low dietary vitamin D intake and rare use of supplements) (54). The possible reason that the associations were more significant with vitamin D insufficiency than with deficiency (Table 4) is that the number of subjects included in the deficient group was much lower than in the insufficiency group.

The *E*-value is the minimum strength of association (scaled by the risk ratio) between unmeasured confounders and treatments and outcomes in conditions where confounders have been measured to fully explain the association between a given treatment and outcome. Simply put, this means that, controlling for measured confounders, if the unmeasured confounding effect wants to completely erase the association effect between exposure and outcome in our study, then the unmeasured confounding effect should be minimized to this value in order to achieve this goal. So, even if there are covariates that are not considered for various reasons, the possibility of unaccounted confounding factors is low as reflected by the *E*-values (26).

There are several limitations in our study. Firstly, this was a cross-sectional study, which precludes comments on the causal impact of vitamin D on general and central obesity in adolescents. Secondly, some variables were not considered due to unavailability across surveys, such as intake of vitamin D supplement, sedentary behavior, sun exposure, parental genetics, and fat indicators in body composition. As reflected by the *E*-values, the possibility of unaccounted confounding factors was relatively low. Thirdly, vitamin D intake was collected by two 24-h dietary recalls, which may not reflect the habitual intake. Fourthly, only adolescents aged 12–18 years from US were included, and whether our findings can be extrapolated to the other age intervals and other races should be made with caution.

Despite these limitations, our findings indicate that deficient or insufficient 25-hydroxyvitamin D concentrations were associated with the significant risk of general and central obesity among US adolescents, and approximately 30% and 50% of these associations, respectively, were mediated by HOMA-IR. More stratified analyses were presented, and identified that the association with general obesity was particularly present among Mexican American, while with central obesity among Non-Hispanic Black adolescents.

## Data Availability

The original contributions presented in the study are included in the article/**Supplementary Material**, further inquiries can be directed to the corresponding authors.
