# Supplementary material for: Serum vitamin D and obesity among US adolescents, NHANES 2011–2018
Source: Front Pediatr. 2024 May 21;12:1334139. doi: 10.3389/fped.2024.1334139 (PMC11148364; doi:10.3389/fped.2024.1334139)
Supplement: Supplementary file 1 [file Presentation1.pdf]

Supplementary Material

Serum Vitamin D and Obesity among US Adolescents, NHANES 2011-2018

Zisu Chen[1,*], Qin Hui[2,*], Xiaojin Qiu[1], Qiong Wang[2,3], Jing Wu[4,#], Min Li[1,#], Wenquan Niu[4,#]

#Correspondence: wujing20221120@163.com (J.W.) or limin@bjzhongyi.com (M.L.) or niuwenquan_shcn@163.com (W.N.).

1 Supplementary Figures

Supplementary Figure 1. Estimated probabilities of general obesity indexed by body mass index (BMI) (panel A) and central obesity indexed by waist circumference to height ratio (WHtR) (panel B) with serum 25-hydroxyvitamin D by confounding factors.

A

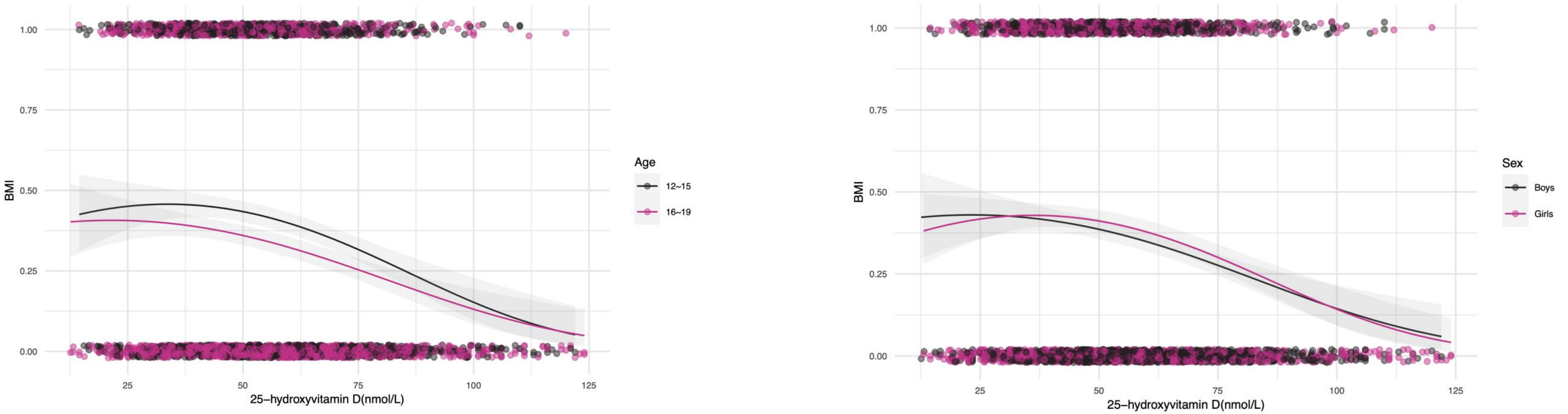

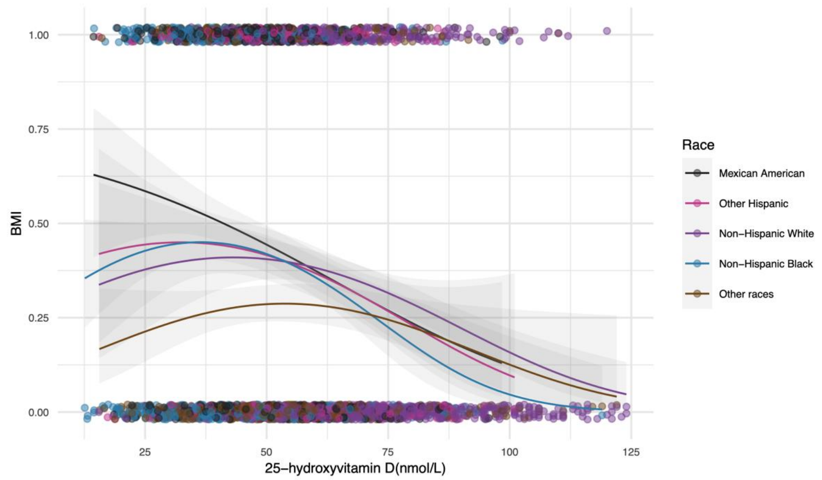

BMI
1.00
0.75
0.50
0.25
0.00
25
50
75
100
125
25−hydroxyvitamin D(nmol/L)
Race
Mexican American
Other Hispanic
Non−Hispanic White
Non−Hispanic Black
Other races

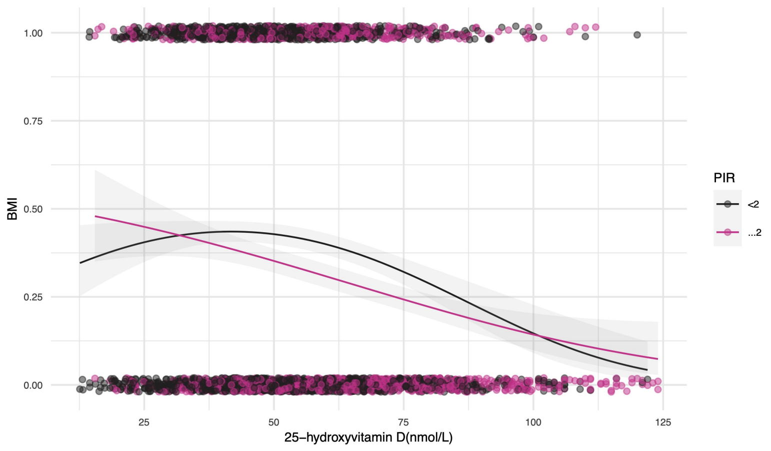

BMI
1.00
0.75
0.50
0.25
0.00
25
50
75
100
125
25−hydroxyvitamin D(nmol/L)
PIR
<2
...2

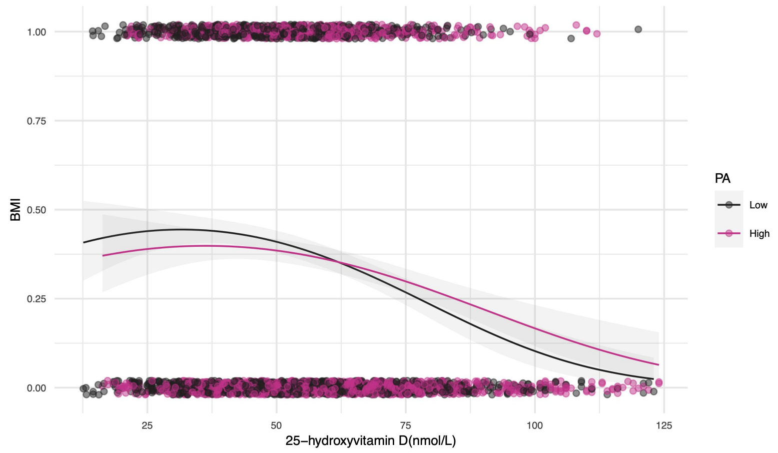

BMI
1.00
0.75
0.50
0.25
0.00
25
50
75
100
125
25−hydroxyvitamin D(nmol/L)
PA
Low
High

B

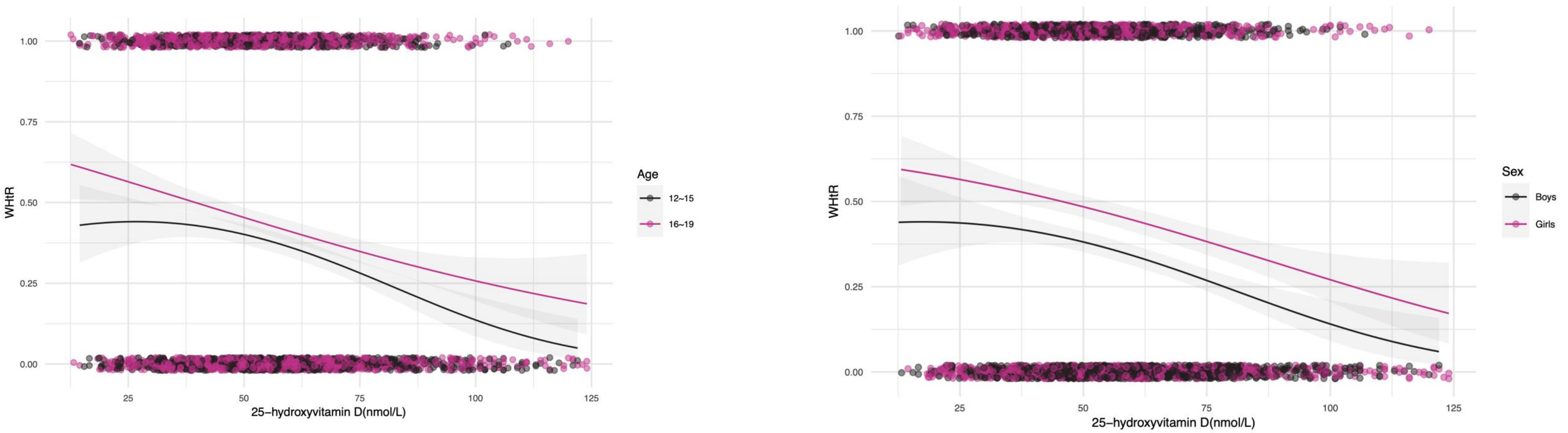
WHtR
1.00
0.75
0.50
0.25
0.00
25
50
75
100
125
25−hydroxyvitamin D(nmol/L)
Age
12~15
16~19
WHtR
1.00
0.75
0.50
0.25
0.00
25
50
75
100
125
25−hydroxyvitamin D(nmol/L)
Sex
Boys
Girls

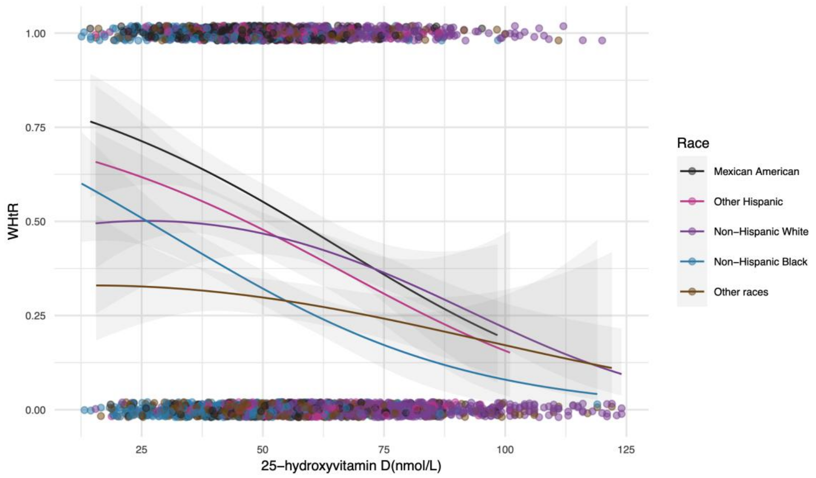

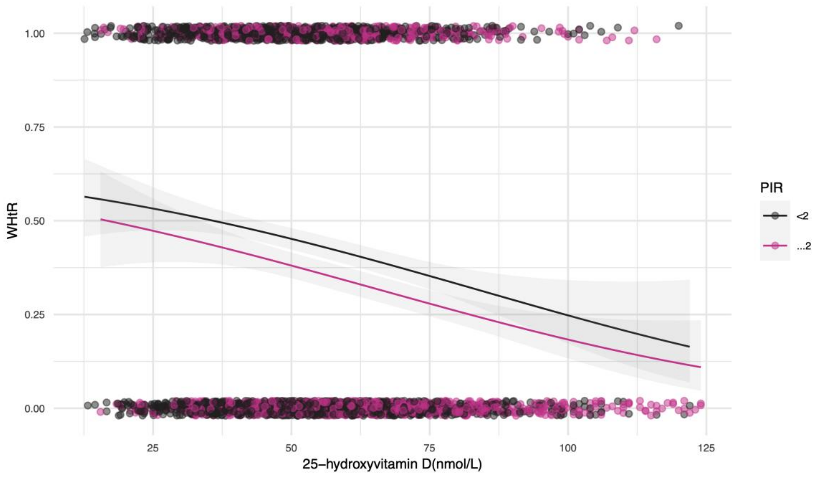

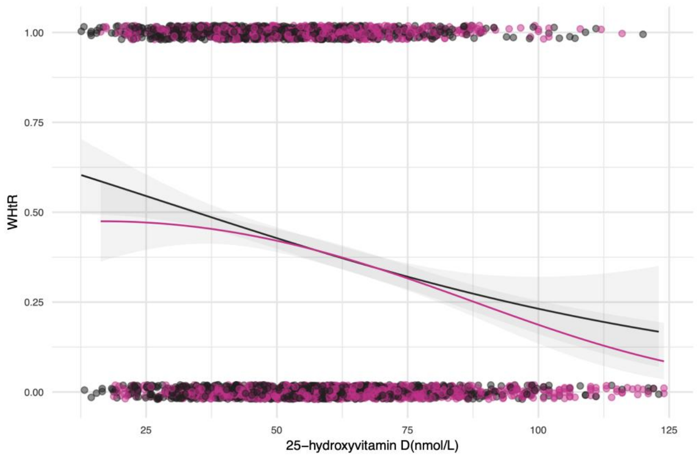

Abbreviations: PIR, poverty income ratio; PA, physical activity.

2 Supplementary analyses - Tables S1 and S2

In order to enrich the original research of our main manuscript, we aimed to assess whether general/central obesity was associated with serum 25-hydroxyvitamin D among US adolescents, and further to explore the mediatory impact of HOMA-IR on this association.

Methods

All data sources are the same as in the main manuscript. The classification of Serum 25-hydroxyvitamin D are based on the Centers for Disease Control (1). Vitamin D deficiency and insufficiency was merged to define vitamin D deficiency (<50nmol/L). General and central obesity were same in the main manuscript. HOMA-IR used for the mediation analysis were treated as categorical using the cut-offs for age and gender (2).

The statistical analysis were the same as in the main manuscript. The association of general and central obesity on a categorical scale with serum 25-hydroxyvitamin D was assessed using the Logistic regression analyse. The Sobel-Goodman mediation test was used to examine whether HOMA-IR can mediate the association of general and central obesity with 25-hydroxyvitamin D. Vitamin D, HOMA-IR, general and central obesity were all treated as categorical.

Results

Table S1 shows the association of general and central obesity with serum 25-hydroxyvitamin D. Taking adolescents with Non-obesity as a reference, the risk for Vitamin D Deficiency was significantly increased in adolescents with general and central obesity before and after adjusting for confounding factors. In adolescents with general obesity, fully-adjusted OR associated with Vitamin D Deficiency was 1.550 (95% CI: 1.294 to 1.856), with central obesity was 1.639 (1.364 to 1.968), respectively.

Table S1. Association of general and central obesity with 25-hydroxyvitamin D.

		Vitamin D Deficiency		
		**Model 1**	**Model 2**	**Model 3**
	Non-obesity	ref.	ref.	ref.
Obesity	General Obesity	1.621 (1.382, 1.901)***	1.575 (1.320, 1.880)***	1.550 (1.294, 1.856)***
	Central Obesity	1.662 (1.418, 1.949)***	1.696 (1.416, 2.031)***	1.639 (1.364, 1.968)***

Abbreviations: Ref., reference. Data are represented as odds ratio (95% confidence interval).
Model 1: no adjustment.
Model 2: adjustment for age, sex, race/ethnicity, and poverty income ratio.
Model 3: adjustment for age, sex, race/ethnicity, poverty income ratio, vitamin D intake, energy, physical activity.
$^{*}p < 0.05$, $^{**}p < 0.01$, $^{***}p < 0.001$.

Provided in **Table S2** is the mediation effect of HOMA-IR on the association of general and central obesity with serum 25-hydroxyvitamin D. Total, natural direct, and natural indirect effects were explored, with statistical significance at a level of 5%. The proportion mediated by HOMA-IR reached as high as 28.6% for global obesity

and 36.6% for central obesity.

Table S2. Mediatory effect of HOMA-IR on the association of general and central obesity with serum 25-hydroxyvitamin D.

Items	Statistics	General Obesity	Central Obesity
Total effect	β	0.085	0.074
	Lower	0.029	0.193
	Upper	0.140	0.129
	P	<0.01	<0.01
Natural direct effect	β	0.060	0.047
	Lower	-0.001	-0.011
	Upper	0.121	0.105
	P	0.051	0.111
Natural indirect effect	β	0.024	0.027
	Lower	-0.004	0.005
	Upper	0.052	0.049
	P	0.094	<0.05
Proportion eliminated		28.6%	36.6%
P value		0.059	0.039

Abbreviations: HOMA-IR, Homeostatic Model Assessment of Insulin Resistance.
P was calculated after adjusting for age, sex, race/ethnicity, poverty income ratio, vitamin D intake, energy, physical activity.

References

[1] Institute of Medicine. Dietary reference intakes for calcium and vitamin D. Washington, DC: National Academies Press. 2010.

[2] Andrade MI, Oliveira JS, Leal VS, Lima NM, Costa EC, Aquino NB, Lira PI. Identificação dos pontos de corte do índice Homeostatic Model Assessment for Insulin Resistance em adolescentes: revisão sistemática [Identification of cutoff points for Homeostatic Model Assessment for Insulin Resistance index in adolescents: systematic review]. Rev Paul Pediatr. 2016 Jun;34(2):234-42. doi: 10.1016/j.rpped.2015.08.006. Epub 2015 Oct 20. PMID: 26559605; PMCID: PMC4917276.
